# Structure Analysis and Its Correlation with Mechanical Properties of Microcellular Polyamide Composites Reinforced with Glass Fibers

**DOI:** 10.3390/ma16237501

**Published:** 2023-12-04

**Authors:** Piotr Szewczykowski, Dariusz Sykutera, Piotr Czyżewski, Mieczysław Cieszko, Zbigniew Szczepański, Bartosz Nowinka

**Affiliations:** 1 Department of Manufacturing Techniques, Faculty of Mechanical Engineering, Bydgoszcz University of Science and Technology, Kaliskiego 7, 85-796 Bydgoszcz, Poland; dariusz.sykutera@pbs.edu.pl (D.S.); piotr.czyzewski@pbs.edu.pl (P.C.); bartosz.nowinka@pbs.edu.pl (B.N.); 2 Department of Mechanics of Porous Media, Faculty of Mechatronics, Kazimierz Wielki University, Chodkiewicza 30, 85-064 Bydgoszcz, Poland; cieszko@ukw.edu.pl (M.C.); zszczep@ukw.edu.pl (Z.S.)

**Keywords:** polymer–matrix composites, mechanical properties, porous materials, X-ray computed microtomography, numerical simulations

## Abstract

Thin-walled and thick-walled microcellular moldings were obtained by MuCell^®^ technology with nitrogen as a supercritical fluid. 2 mm thick polyamide 6 (PA6) with 30% wt. glass fiber (GF) samples were cut from automotive industrial elements, while 4 mm, 6 mm, and 8.4 mm thick moldings of PA6.6 with 30% wt. GF were molded into a dumbbell shape. The internal structure was investigated by scanning electron microscopy (SEM) and X-ray computed microtomography (micro-CT) and compared by numerical simulations for microcellular moldings using Moldex3D^®^ 2022 software. Young’s modulus, and tensile and impact strength were investigated. Weak mechanical properties of 2 mm thick samples and excellent results for thick-walled moldings were explained. SEM pictures, micro-CT, and simulation graphs revealed the tendency to decrease the cell size diameter together with increasing sample thickness from 2 mm up to 8.4 mm.

## 1. Introduction

The global industrial tendency is to reduce the mass of all means of transport to reduce carbon dioxide and other greenhouse gas emissions. Although common thermoplastic materials are being criticized for their influence on the environment after their life cycle, there are still no better recyclable materials than polymer composites with a much lower density and comparable strength to metals. Reinforced engineering thermoplastics are often an alternative material for aluminum and steel because of their high strength, modulus, chemical, and thermal resistance. According to the Plastics Europe report, the European automotive industry is the third biggest end-use market with 8.6% of the total demand for European plastics converters, which is 50.3 million tons (in full) in the year 2022 [1]. Polypropylene (PP) and polyamides (PA) are the automotive industry’s most applied thermoplastics. Long and short glass and carbon fibers can reinforce the mechanical strength of both materials, which negatively influences their density and processability. Therefore, applying chemical blowing agents [2,3] or physical processes like MuCell^®^, IQ Foam^®^ or Ku-FizzTM enables obtaining microcellular structures, causing mass reduction [4,5]. Numerous microcellular polymers and composites like polyolefins, polycarbonates, and polyesters find their applications in the automotive industry and other industries [6,7]. Fibers can act as heterogeneous cell nucleants, reducing cell diameter and increasing cells density in a volume, which leads to a uniform cellular structure and better mechanical properties of products [8]. Since fiber orientation, cell diameter, and distribution influence products’ mechanical properties, there is interest in investigating materials’ internal structure [9]. 

Sample preparation either for SEM or optical microscopy is not straightforward with thick-walled (thicker than 4 mm) samples of polyamides reinforced with glass fiber [10]. Because of cell deformation, the invasive sample preparation process can be even more difficult for foamed pieces. Therefore, X-ray computed microtomography (micro-CT) is becoming a more popular non-destructive method of internal structure observations. Many radiographic projections, called shadow projections, are captured at different angular positions, transversal cross-sections (slices) are reconstructed, and the 3D analysis of the investigated material can be performed. Even though sample preparation does not require special treatment, there are many difficulties in proper image reconstruction and the interpretation of results, especially in systems with three phases: polymeric matrix, pores filled with gas, and glass fibers [11]. There are numerous X-ray computed microtomography in situ investigations of fatigue tests of solid PA6 and PA66 reinforced with glass fibers [12,13,14,15,16,17,18] or ex situ observations of glass fibers [19,20,21] and woven textiles [22,23,24]. The evolution of voids in non-reinforced PA during tensile tests was investigated by micro-CT as well [25,26]. In most cases above, articles present results obtained by synchrotron radiation, which gives a much better X-ray beam than commercial microtomography, resulting in higher resolution (0.65–0.7 µm), no need for beam hardening, and minor artifacts. SEM and micro-CT techniques reveal a typical skin–shell–core structure in the case of PA reinforced with glass fibers [8,13]. In contrast, the microcellular form has an additional transition layer [8,27,28], which causes such a three-phase structure to be even more complicated to investigate using the commercial micro-CT apparatus applied in this research. 

It should be stressed that most publications about foamed polyamide reinforced with GF describe moldings in the shape of 1.5–4 mm thick plates, which would eventually become standard specimens [8,27,29,30]. Those papers present structural effects depending on the sample’s geometrical features and processing conditions of microcellular injection molding (MIM) technology. The average pore size for samples in this thickness is between 50 and 100 μm [27,29,31], and the number of cells in the PA composite is 2.78–17.2 × 10^7^ cells per cm^3^ [30]. Glass fibers are oriented parallel to the melt flow direction [5,8,30,31,32] in a shell/transition zone while perpendicular in a core region, which applies to different polymer matrixes and fillers [9,33,34]. 

The aim of the work was to determine the influence of the thickness of porous moldings (including molding with a thickness above 4 mm) obtained using the MuCell^®^ method on the distribution and pore size of PA moldings filled with 30% glass fibers. The effect of structural anisotropy on the mechanical properties was examined. In the structural studies, unlike other researchers, a commercial microtomography was used to verify the obtained results, and a comparative analysis was performed in the same cross-sections of the pore distribution using SEM and FEM analysis, and due to the fact that it is difficult to apply to this type of material, it was related to numerical simulation using Moldex3D^®^ 2022 software (CoreTech Systems, Chupei, Taiwan).

## 2. Materials and Methods

Four different sample thicknesses were investigated in this article: 2 mm, 4 mm, 6 mm, and 8.4 mm. A 2 mm thick sample was cut from the large-scale industrial element, w element, while 4 mm, 6 mm, and 8 mm thick samples were obtained in the laboratory injection mold. For clarity, features characterizing each sample are summarized in Table 1. The authors chose samples that, independently of gas-adding parameters, obtained the most similar density reduction (c.a. 5 wt%), which is also included in the below table.

### 2.1. Materials

**Preparation of 2 mm thick samples**

**Figure 1:**
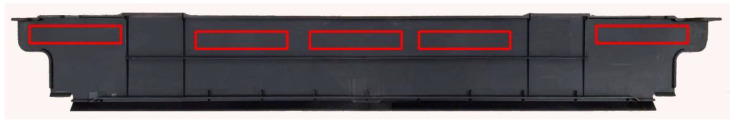
Industrial automotive element with marked places (red frames) from which 2 mm thick samples were cut and analyzed.

Samples in the 1.9–2.1 mm thickness range were cut from industrial elements of 680 × 100 mm dimensions produced for automotive applications (Figure 1). Polyamide 6 Akulon K224-HG6 (DSM, Heerlen, The Netherlands) contained 30 wt% of short glass fibers (GF) and showed 1350 kg/m^3^ density and a melting point of 220 °C. This material was applied to produce industrial elements. Polyamide was dried for 4 h at 105 °C in a vacuum chamber before polymer processing.

**Preparation of 4 mm thick samples**

Polyamide 66 Technyl AR 130GF (Solvay, Brussels, Belgium) with 30 wt% short glass fibers was applied for 4 mm thick samples. The polymer density was 1370 kg/m^3^, and the melting point was 260 °C. Polyamide was dried for 4 h at 80 °C in a vacuum chamber before processing.

**Preparation of 6 mm and 8.4 mm thick samples**

Both 6 mm and 8.4 mm samples were prepared from polyamide PA66 GF30 Technyl AR 130/1 (Rhodia, La Defence, France) containing 30 wt% of short glass fiber. Polyamide density was 1370 kg/m^3^, and the melting point was 263 °C. Before polymer processing, the material was dried for 4 h in a vacuum chamber at 80 °C.

Detailed information about applied materials is presented in Table 2.

### 2.2. Testing Specimen and Sample Preparation

#### 2.2.1. Foamed Specimen Preparation

**Figure 2:**
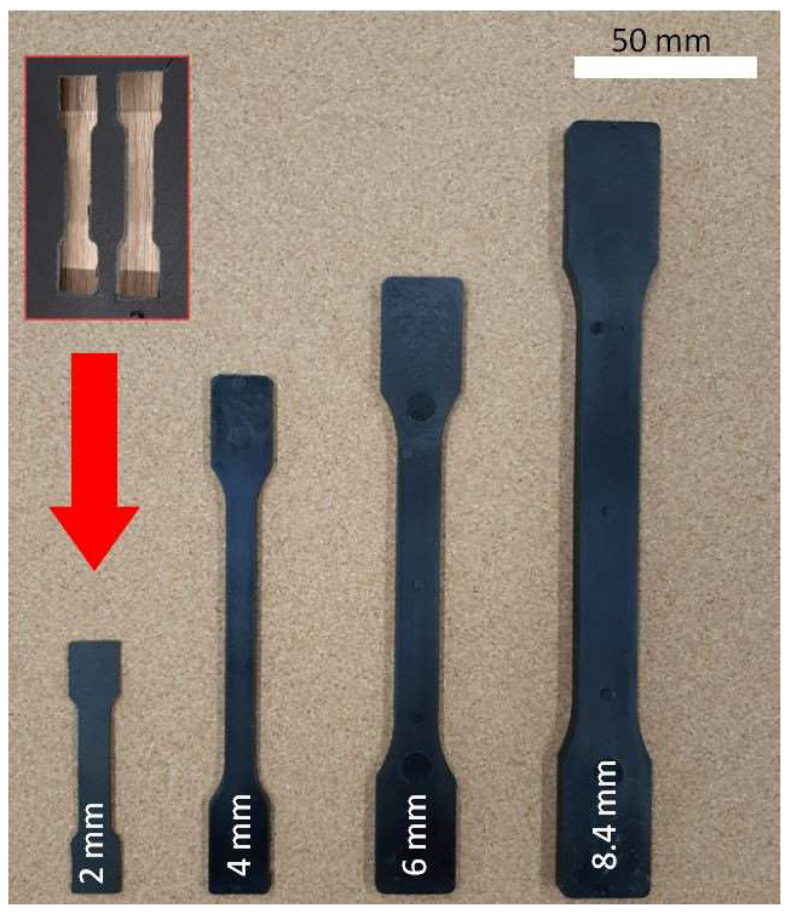
Tensile testing samples at four different thicknesses: 2 mm; 4 mm; 6 mm, and 8.4 mm (from left to right). 2 mm thick samples were cut from the industrial element (red frame and arrow) while 4 mm; 6 mm and 8 mm thick were directly injection molded.

Since four different moldings are injected under different mold and processing parameters, the essential processing parameters are summarized in Table 3 to clarify the differences between the samples. The obtained moldings had a surface free of sink marks on the surface (Figure 2).

**Molding of 2 mm thick samples**

Large-scale industrial elements were obtained via the microcellular injection molding process at the Graform company (Bydgoszcz, Poland). An Engel Victory 500 (Engel, Schwertberg, Austria) injection molding machine and MuCell^®^ technology (Trexel, Wilmington, MA, USA) were used in this process. Injection molding parameters are summarized in Table 3. A hydraulic press was used to cut tensile test specimens from large-scale elements according to PN-EN ISO 527-2:2012 [35].

**Molding of 4 mm thick samples**

The MuCell^®^ process was carried out in a four-cavity laboratory injection mold to produce tensile testing samples according to the PN-EN ISO 527-2:2012 [35] standard. 16 × 2.1 mm gap gates were applied. A hydraulic Victory 500 injection molding machine (Engel, Schwertberg, Austria) was used in the microporous injection tests. Injection molding parameters are summarized in Table 3.

**Molding of 6 mm and 8.4 mm thick samples**

6 mm and 8.4 mm molded pieces, here described as thick-walled samples, were produced using an Engel Victory 500 (Engel, Schwertberg, Austria) injection molding machine, in a four-cavity injection mold [28]. MuCell^®^ technology (Trexel, Wilmington, MA, USA) was applied as a microcellular injection process. The dimensions of obtained 6 mm and 8.4 mm thick molded pieces were 1.5 and 2 times higher compared to PN-EN ISO 527-2:2012 [35] type 1B samples, respectively. 24 × 4.2 mm and 21 × 4.1 mm (wide × deep) gap gates were applied for 8.4 mm and 6 mm specimens, respectively, in the mold cavity [28]. Injection molding parameters are summarized in Table 3. 

#### 2.2.2. Sample Preparation for SEM and Micro CT Analysis

**Figure 3:**
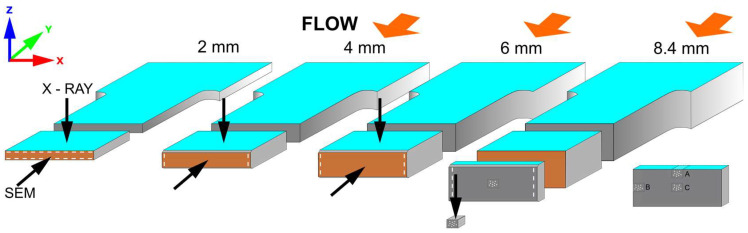
Sample preparation for SEM and X-ray micro-CT of samples with different thicknesses. Black arrows show surfaces investigated by SEM and X-ray beam hitting the sample. Observed skin layers are marked with a white dashed line. Smaller pieces were additionally cut from the core and edges of 8.4 mm thick sample, C and A, B, respectively. Flow direction is marked, except for a 2 mm thick sample, for which a polymer melt flow is explained in the other part of the article.

The structural properties, mainly pore size and its distributions were investigated by Scanning Electron Microscopy (SEM) (JEOL, Tokyo, Japan) and X-ray computed microtomography (micro-CT) (Bruker, Kontich, Belgium) images. All images were taken from the central part of the measurement zone. Samples were prepared for SEM analysis by mechanical breaking after keeping the sample for 1 min in liquid nitrogen. To obtain a flat, undeformed surface for SEM, samples of 4 mm, 6 mm, and 8.4 mm thickness were notched on the longer sides before being hit and broken. Therefore, the skin layer of those thick samples was analyzed on the short edges, which are marked with white, dashed lines in Figure 3. The 2 mm samples were cut from a large element, so it was not possible to examine the skin on the shorter side. Sample surfaces were sputter-coated with platinum for 30 s before being placed in the SEM chamber. X-ray computed microtomography was used to investigate the pieces, with the X-ray beam perpendicular to the polymer flow direction. For an 8.4 mm thick sample, a piece of a size of c.a. 1 mm^3^ was cut to obtain higher-resolution images (Figure 3).

### 2.3. Measurements of Density and Mechanical Properties

Mechanical properties of all samples were investigated using a tensile test machine Z030 (Zwick/Roell, Ulm, Germany) according to PN-EN ISO 527-2:2012 [35]. The machine is equipped with a measuring head with a load capacity of 30 kN. The impact strength was measured for all samples at 23 °C according to PN-EN ISO 179-2:2020-12 [36] (Charpy impact test) by using a HIT50 Pendulum Impact Tester (Zwick/Roell, Germany) with a pendulum of 25 J or 50 J (in case of 8.4 mm samples thickness). For 8.4 mm thick samples the fracture took place at the shorter edge. The density of the samples was measured by a hydrostatic method with methanol as the immersion liquid. AD50 (Axis, Poland) laboratory scales were used. 

### 2.4. SEM and Micro-CT Analysis

The sample surface was investigated using a JEOL 5600 electron microscope (JEOL, Tokyo, Japan) at 1 kV acceleration voltage after sputter-coating with a platinum layer. Each sample was scanned from the left to the right edge, and pictures of 35× and 150× magnification were joined together in order to estimate the thickness of the skin layer and average pore size and pore size distribution. Images were analyzed by using ImageJ 1.53k software (Rasband, W.S., ImageJ, U. S. National Institutes of Health, Bethesda, MD, USA). 

A sample of 2 mm thickness was investigated by Bruker SkySkan 1173 X-ray micro computer tomography (Kontich, Belgium), while 4 mm, 6 mm, and 8.4 mm samples were analyzed by Bruker SkyScan 1272 X-ray micro-computed tomography (Kontich, Belgium). Projections were reconstructed by the NRecon program, the analysis was performed using the CT Analyzer program, while the 2D and 3D visualization of glass fiber orientation was obtained by DataViewer and CTvox programs by Bruker. 

### 2.5. Moldex3D^®^ Simulation of MIM Process

The Foam Injection Molding module by Moldex3D^®^ (CoreTech System Co., Ltd., Chupei, Taiwan) software was used to calculate an average cell size and cell density. The selection of this software was based on previous experiences with FEM analysis of pore growth [37]. Injection molding process parameters used for simulation conduction correspond to the conditions used for experimental samples. The bubble growth model used to predict the porous structure of the sample was the Han and Yoo model. The mesh parameters used to convert 3D models to finite elements are presented in Table 4. To obtain reliable results, the criterion was adopted that further increasing the number of finite elements did not significantly affect the simulation results. On this basis, the size of the mesh was determined. The analyzed results were read with the use of eleven measurement points distributed evenly through the thickness of the analyzed part. Depending on the element’s geometry, the distance between successive measurement points was equal to 10% of the part’s thickness.

## 3. Results and Discussion

### 3.1. SEM Pictures Analysis

**Analysis of 2 mm thick samples**

**Figure 4:**
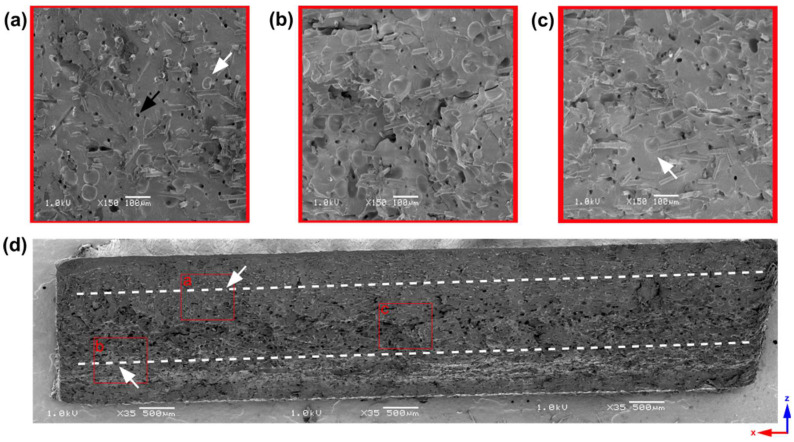
Scanning electron microscopy pictures of a 2 mm thick sample. White dashed lines show the distance from the sample edge to the first cells (marked by white arrows) observed by SEM. The chosen regions of the sample cross-section (**d**) at the upper skin layer (**a**), bottom skin layer (**b**), and a sample core (**c**) at 150× magnification are marked with red frames. The empty hole after removing the single glass fiber is marked with a black arrow (**a**).

In Figure 4, a scanning electron microscopy picture shows a 2 mm thick sample cross-section. A white dashed line shows the skin layer, a distance between the sample edge and the first pores observed by SEM. One side’s skin layer is about 500 µm, 25% of the total sample thickness. The upper pictures present 150x magnification, with clearly seen pores of an average size of 56.21 ± 10.61 µm based on ImageJ software analysis. Throughout the cross-section of the core part, many pores with a size of up to 85 µm are visible and can act as notches during mechanical properties investigations. Compared to pore diameters of other polyamide samples reported in the literature, these are the large-sized pores existing in thin-wall moldings, which can partly explain the weak mechanical properties of 2 mm thick samples [27,29]. The black arrow (Figure 4a) marks the hole after removing a glass fiber, whose dark color, regular shape, and diameter distinguish it from a pore marked with a white arrow.

**Analysis of 4 mm thick samples**

**Figure 5:**
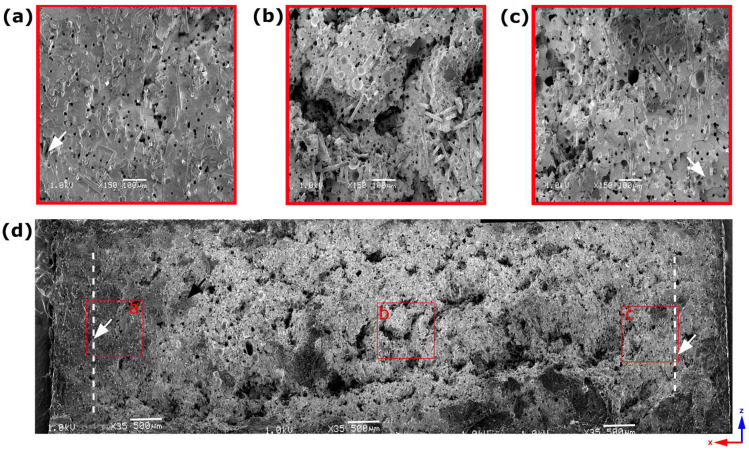
Scanning electron microscopy pictures of a 4 mm thick sample. White dashed lines show the distance from the sample edge to the first cells (marked by white arrows) observed by SEM. The chosen regions of the sample cross-section (**d**) at the left-edge skin layer (**a**), right-edge skin layer (**c**), and a sample core (**b**) at 150× magnification are marked with red frames.

In the case of a 4 mm thick sample, the observed skin layer (Figure 5) is around 700 µm. Comparing it to the sample width (10 mm), it is about 7%. Based on ImageJ 1.53k software analysis, the average pore size is 18.41 ± 9.06, µm which means that for a twice-as-thick sample (compared to 2 mm thick), pores are 67.25% smaller. Regions within the skin layer show better glass fiber orientation in the flow direction (y-axis) (Figure 5a,c) compared to more randomly oriented fibers in a core (Figure 5b).

**Analysis of 6 mm thick samples**

**Figure 6:**
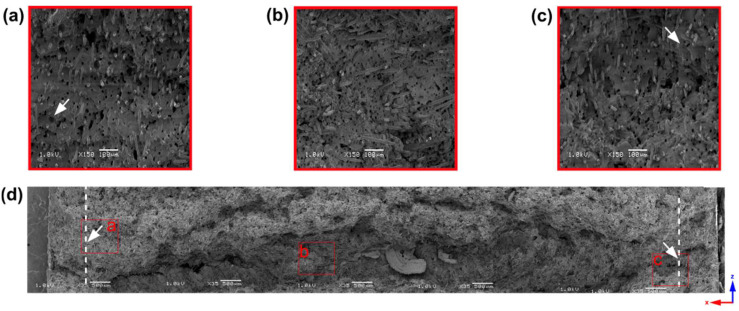
Scanning electron microscopy pictures of a 6 mm thick sample. White dashed lines show the distance from the sample edge to the first cells (white arrows) observed by SEM. The chosen regions of the sample cross-section (**d**) at the left-edge skin layer (**a**), right-edge skin layer (**c**), and a sample core (**b**) at 150× magnification are marked with red frames.

A scanning electron microscopy cross-section for a 6 mm thick sample is shown in Figure 6. The skin layer, meaning the distance to the first observed cells, is estimated to be around 800 µm, compared to the sample width of 15 mm, giving 5.3% on each side. At 150× magnification, fiber orientation can be observed even better in the range of a skin layer (Figure 6a,c). In contrast, more chaotic fibers positioning can be seen in the core region of a sample (Figure 6b). The average pore size based on ImageJ 1.53k software analysis is 15.73 ± 4.13 µm.

**Analysis of 8.4 mm thick samples**

**Figure 7:**
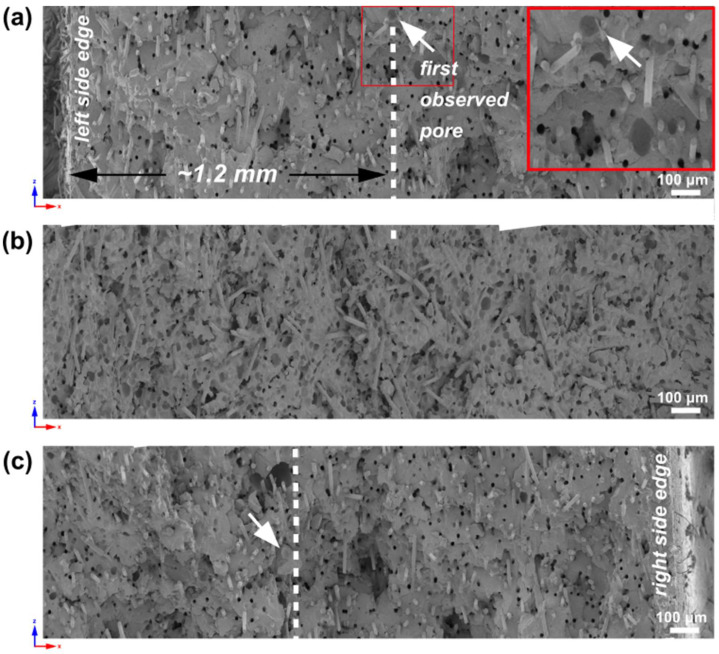
Scanning electron microscopy pictures of 8.4 mm thick sample’s region from the left edge (**a**), core (**b**), and right edge (**c**). White dashed lines show the distance from the sample edge to the first noticed pores (white arrows) observed by SEM. The first observed pore from the left-side sample edge is magnified in a red-framed picture.

Due to the height (18.6 mm) and width of the 8.4 mm thick sample, the combined SEM pictures of the whole sample cross-section was challenging to present in one image, so only regions of a skin layer on both sides and core region are shown in Figure 7a, Figure 7c and Figure 7b, respectively. The thickness of a skin layer estimated as 1.2 mm is the highest compared to other samples and, compared to the sample width of 20 mm, gives 6% on each side. SEM pictures reveal fiber orientation along the polymer melt flow within the skin layer (Figure 7a,c), while fiber disorientation is observed in the core part (Figure 7b). The average pore size is slightly higher compared to the 6 mm thick sample, which is 17.98 ± 5.08 µm.

**Figure 8:**
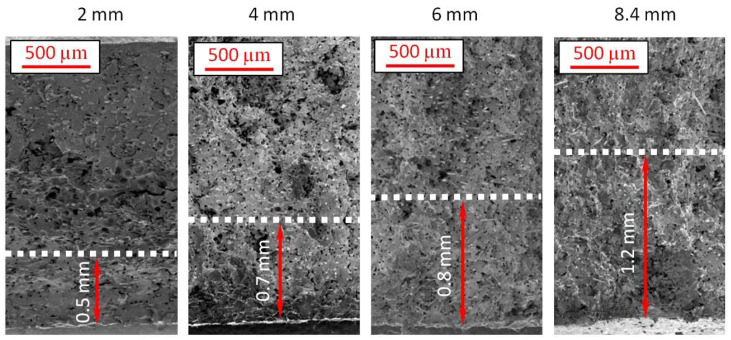
Comparison of skin layer thickness for different samples with marked distance from the edge to the first cells observed by SEM.

Based on previous analyses and observations, a reasonably wide transition zone for 6 mm and 8.4 mm thick samples shows glass fibers oriented in the flow direction [28,38]. A comparison of sample skin layers is presented in Figure 8, showing increasing skin layer thickness for 2 mm, 4 mm, 6 mm, and 8.4 mm thick pieces. The thinnest 2 mm sample has a relatively larger skin-layer thickness compared to its overall thickness (approx. 25% for one side). The skin layer’s thickness increase does not correlate with the moldings’ thickness change. The fastest cooling rate for a molded part’s cross-section is seen in a 2 mm part, resulting in the lowest likelihood of forming a fine-pored structure. Four-times-thicker molding, compared to a 2 mm thick one, reveals only two-times-higher skin-layer thickness with no cells.

**Figure 9:**
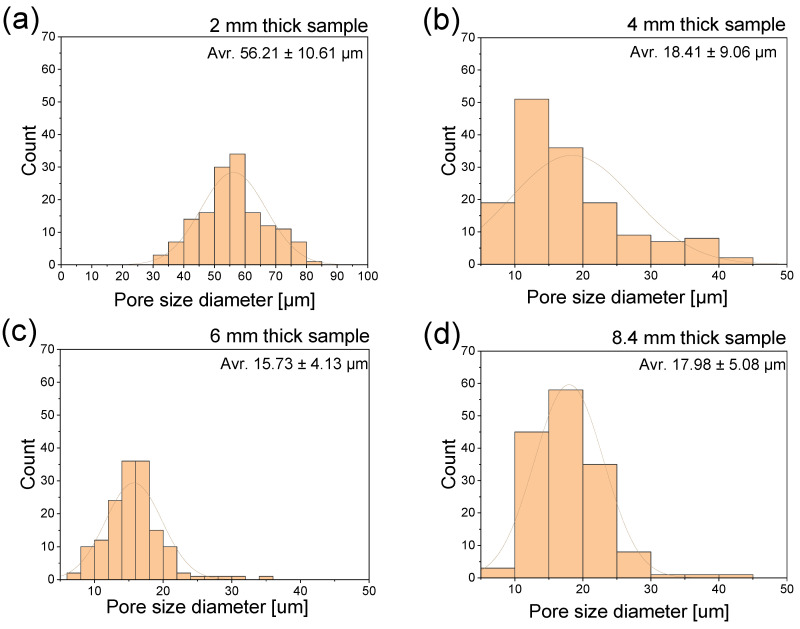
Histograms of pore size diameter for 2 mm (**a**), 4 mm (**b**), 6 mm (**c**) and 8.4 mm (**d**) sample thicknesses based on the SEM pictures analyzed by ImageJ software. Comparison of sample skin thickness for different samples. Average pore size values are marked in the upper right corners.

Figure 9 shows histograms of pore size diameter based on ImageJ 1.53k software analysis of SEM pictures. The analysis was performed manually by exploiting the presence of clearly recognizable cells. The most frequent pores for a 2 mm thick sample are within the 50–60 µm diameter, and the maximum observed size reaches 85 µm. It was hard to indicate pores smaller than 30 µm. In all the other samples’ thicknesses, there is a fraction of tiny pores below 10 µm, which is close to or even less than glass fiber diameter. Pictures of 4 mm, 6 mm, and 8.4 mm thick samples reveal a maximum pore size within the 35–45 µm range, which shows a considerable difference compared to 2 mm thick samples.

### 3.2. X-ray Microtomography Analysis

Comparing micro-CT to SEM results, the tendency for average pore size depending on sample thickness is similar. The highest, 32.59 µm pore diameter values are for 2 mm thick sample, and 28.37 µm, 16.87 µm, and 23.95 µm diameter for 4 mm, 6 mm, and 8.4 mm samples, respectively. Values from micro-CT are single results without std dev since this is the result generated by CTAn software, version 1.16.4.1 after the 3D analysis option.

**Figure 10:**
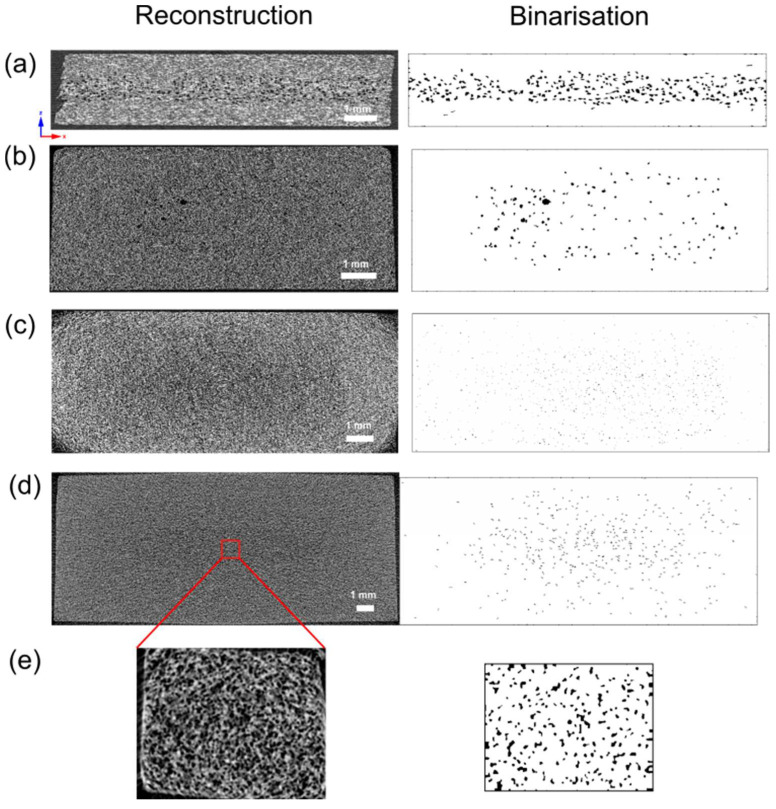
Single cross-sections of 2 mm (**a**), 4 mm (**b**), 6 mm (**c**) and 8.4 mm (**d**) sample thicknesses after X-ray microtomography data reconstruction (left column) and binarization (right column). 8.4 mm thickness sample was additionally analyzed after cutting a 1 mm^3^ piece from the sample core (**e**).

Figure 10 presents reconstruction layers in the left column and binarized pictures in a right column. The reconstructed images show the whole cross-section of a sample before choosing the region of interest (ROI) for further binarization. The ROI was selected as close as possible to the original sample edge. For the 8.4 mm thick sample, an additional piece was cut from the sample core and analyzed at a higher resolution, as presented in Figure 10e.

The critical point in the resulting data from micro-CT is, first of all, a scanning procedure and resolution. For all samples, the authors tried to scan the entirety of all samples to observe the entire spectrum of a cross-section, but the resolution was low, ranging from 11 µm image pixel size up to 5 µm. 

In CTAn software, the higher the resolution, the more accessible it was to cut the pores from the background [39,40]. Those pores close to the glass fiber diameter can be distinguished in the SEM pictures, while binarization makes it more challenging in the CTAn program. Choosing the threshold values is based on comparing the original image after reconstruction with the binarization picture after changing threshold values. The result can be discussed mainly for low-resolution images and a lot of noise. 

A thresholding method is essential and critical in obtaining accurate results [39,41]. This method divides an image into two phases: cells and matrix. Therefore, it is challenging to distinguish glass fiber, especially with a low contrast between matrix and glass and low resolution. The thresh-holding method can produce a lot of noise, which may mistakenly be classified as a pore. 

**Figure 11:**
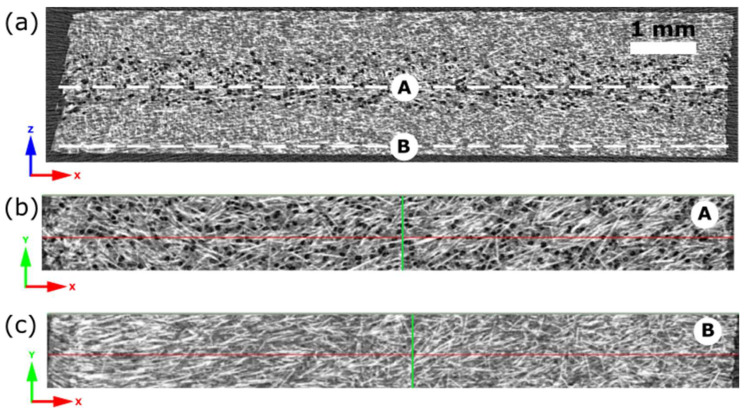
Cross-sections (z-x) for a 2 mm thick sample with visible pores (**a**). Cross-sections along the y-axis at points (A, B) refer to sample core (**b**) and close to the sample surface (**c**).

Example cross-sections of a 2 mm thick sample viewed using the DataViever program by Bruker are shown in Figure 11. The z-axis refers to the thickness of the automotive element. Reconstructed pictures enable us to observe glass fiber orientation in a core (Figure 11b) and close to the sample surface (Figure 11c). A chaotic direction is seen in the center, and similarly, a clear orientation can be challenging to find in cross-sections close to the surface.

**Figure 12:**
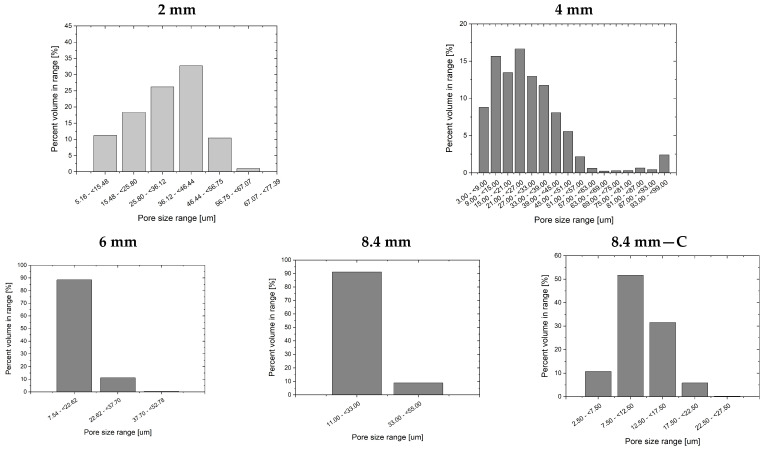
Pore size distributions for different sample thickness based on X-ray microcomputed tomography results. The 8.4 mm thick sample was additionally analyzed for smaller piece cut from the sample core (8.4 mm—C).

Figure 12 presents the pore size distribution resulting from micro-CT analysis, which can be compared to SEM analysis by ImageJ. For the 2 mm thick sample, most pores are within 36–46 µm, which is lower than the corresponding ImageJ results. In the case of a 4 mm thick sample, a 21–27 µm cell size is the dominating value which is 10 µm higher compared to the average SEM picture. The 6 mm and 8.4 mm thick samples are much more uniform. Fractions of 7.54–22.62 mm and 11.00–33.00 mm are c.a. 90% volume for thick pieces. X-ray computed microtomography of a 1 mm^3^ piece cut from the sample core reveals a more accurate pore size distribution (Figure 12c), where c.a. 50% in the volume are pores of 7.5–12.5 µm.

**Figure 13:**
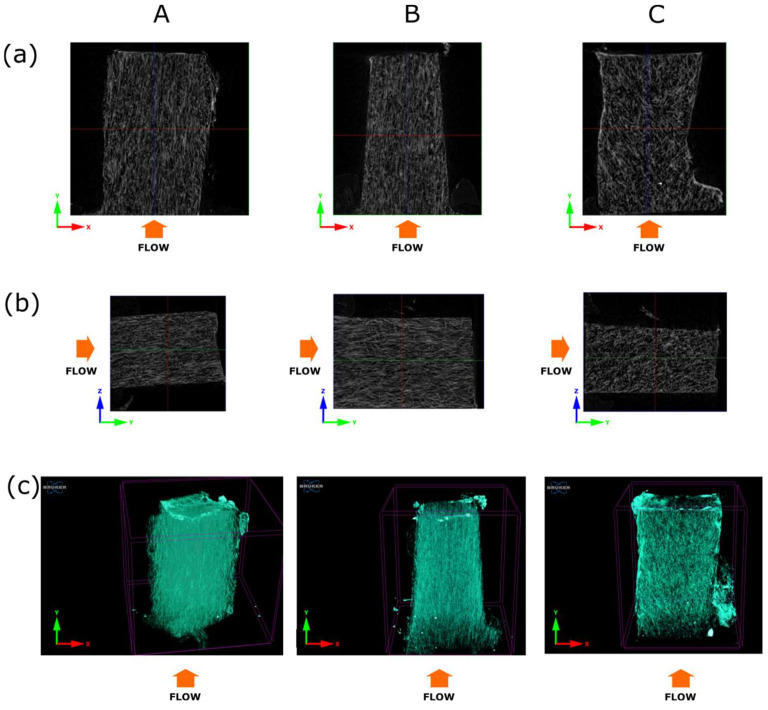
Cross-sections for 8.4 mm thick sample pieces cut from sample edges (column (A) and (B)) and sample core (column (C)). The upper row (**a**) shows the x-z plane, and the middle row (**b**) shows the y-z plane. The bottom row (**c**) presents 3D visualization of the orientation of the fibers.

Three small pieces (c.a. 1 mm^3^) were cut from the sample 8.4 mm thick at the two surfaces, and the sample core and marked as A, B, and C, respectively (Figure 3). 2D and 3D visualizations obtained by DataViewer and CTvox are presented in Figure 13. The orange arrow marks polymer melt flow. The bottom row shows the 3D visualization of all three pieces. Glass fibers are oriented parallel to the melt flow in the case of pieces A and B, while in the core part, fibers are more disoriented.

The distributions and pore sizes confirmed by SEM and micro-CT methods result from several reasons. In the case of samples with the smallest thickness of 2 mm, it should be remembered that they were obtained by cutting from a large-molded part, in places away from injection points. The flowing polymer melt cooled faster, its viscosity increased, and high-shear stresses caused smaller pores to merge into larger, irregular complexes. An additional factor in the formation of an irregular porous structure was the fact that a poreless, quickly solidified skin layer constituted almost 50% of the molded part thickness. The small volume of the material in the melt state did not provide good conditions for the formation of fine and regular cells. 

For the remaining moldings, the pore distribution is similar because the shape of gaps and cavities in the molds are similar. The thickest molded parts (6 and 8.4 mm) were formed in the same injection mold. Therefore, it can be concluded that in this case, the melt flow speed was significantly higher in the case of a smaller-thickness molding, which probably resulted in a reduction in the average size of the gas pores in the structure of these moldings. This is consistent with the effects described in papers [30,42,43]. For this reason, among others, the size of the pores in the 6 mm molding was lower than in the 8.4 mm moldings, which disturbed the linear relationship between the thickness of the molding and the size of the pores. 

### 3.3. Moldex3D^®^ Simulation Results

**Figure 14:**
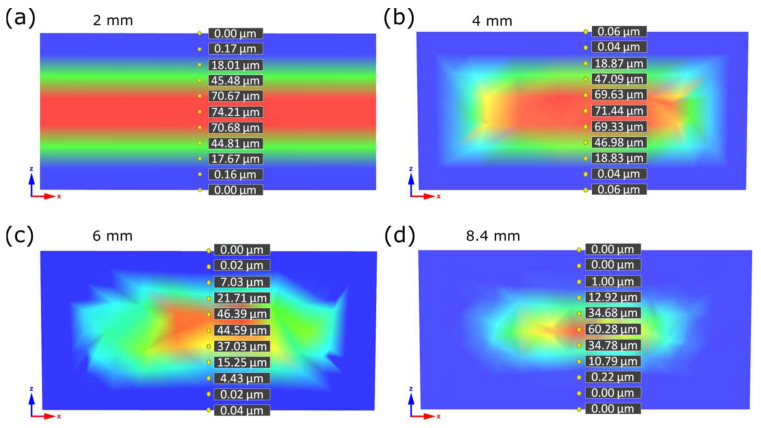
Simulated pore sizes in marked places for 2 mm (**a**), 4 mm (**b**), 6 mm (**c**) and 8.4 mm (**d**) sample thickness along the line from one to opposite sample edge.

Pore sizes for Moldex3D^®^ simulation show much higher results than SEM pictures analysis and micro-CT results (Figure 14). Those results are based on theoretical equations and do not consider all the circumstances occurring in such a complex process as microcellular injection molding. For example, the simulation software does not consider the plasticization phase, which was proved to have a large impact on the microcellular structure and properties of injection molded parts [44,45]. As a result, the pore size calculated by finite elements methods cannot be expected to match the actual values perfectly. However, the tendency in a pore size diameter depending on sample thickness is very similar to the results of micro-CT (Table 5). In all the considered cases, the pore size in the core area of the sample, calculated by Moldex3D^®^, was about 2.5 times larger than the average pore size obtained in the micro-CT analysis. These prove the validity of obtained micro-CT results and the observed pore size values as a function of sample thickness.

**Figure 15:**
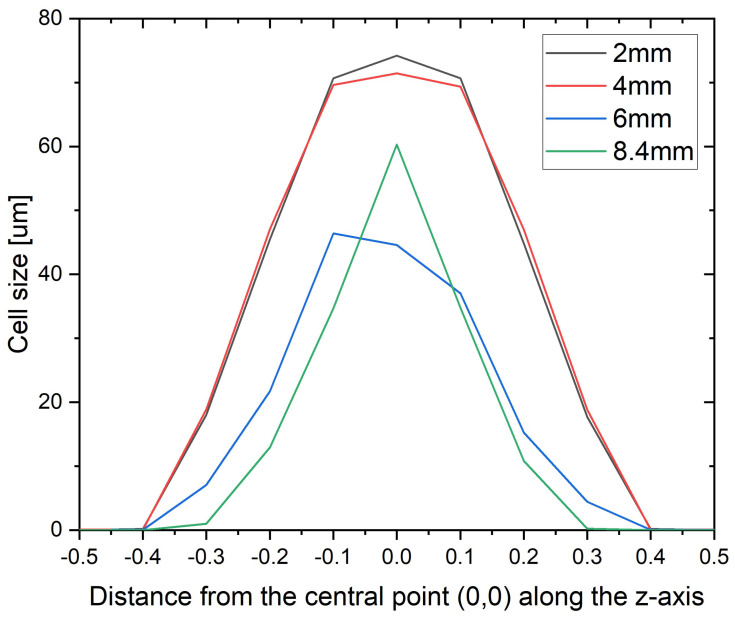
Distribution of a pore size along the Z-axis from the cross-section central point (0%) to the sample edges (−50% and 50%).

Figure 15 shows cell size distribution as a function of distance from the molding’s central point. Regardless of the thickness tested, the largest pores were observed in the core region of the sample. All the curves indicate an upward trend of cell size value measured from the surface to the core region of molding. Based on the simulation results, the finest pores were observed with a 6 mm thick sample. A similar effect was observed on micro-CT and SEM images. All the testing methods observed a corresponding relation between cell size and sample thickness.

**Figure 16:**
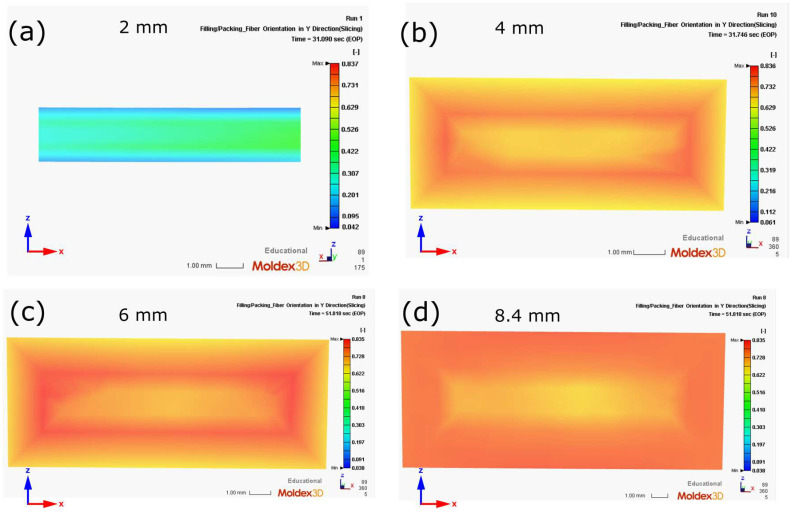
The orientation of the reinforcing fibers in the measuring cross-section of the samples used in the strength tests: (**a**) 2 mm (cut-out cross-section), (**b**) 4 mm, (**c**) 6 mm, (**d**) 8.4 mm (the more orange the color, the greater the orientation of the glass fibers).

The orientation of glass fibers was also numerically analyzed. This factor and the geometry of the porous structure determine the final mechanical properties of injection moldings [4]. Figure 16 shows the fiber orientation toward the longitudinal axis of the test specimens (parallel to the tensile axis), determined by injection simulations. The results can take values from 0 to 1, where the highest value means complete alignment in the considered direction, and 0 means perpendicular alignment to the axis. The results show an unfavorable fiber orientation for a 2 mm thick molding. Here, the maximum orientation value recorded concerning the longitudinal axis was approximately 0.5. With the other samples tested, this parameter reached the value of 0.8. Exceptionally high values of this orientation were observed in the 8.4 mm thick moldings. The strongly oriented fibers in the tensile direction are visible both in the transition and skin layers. This phenomenon was not observed for the thicknesses equal to 4 mm and 6 mm, where the orientation in the skin layer is weaker than in the transition layer. However, a 6 mm thick specimen exhibited a higher fiber orientation than a 4 mm thick specimen.

**Figure 17:**
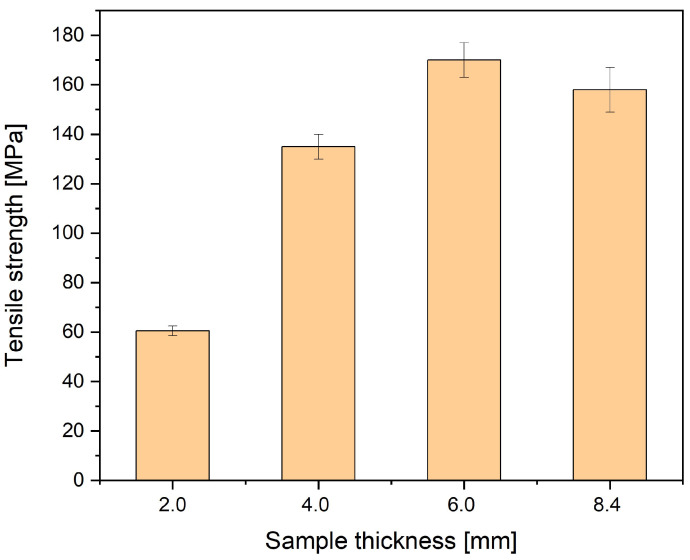
Tensile strength for four samples thicknesses. Values are 61 MPa, 135 MPa, 170 MPa, and 158 MPa for 2 mm, 4 mm, 6 mm, and 8.4 mm thick samples, respectively.

**Figure 18:**
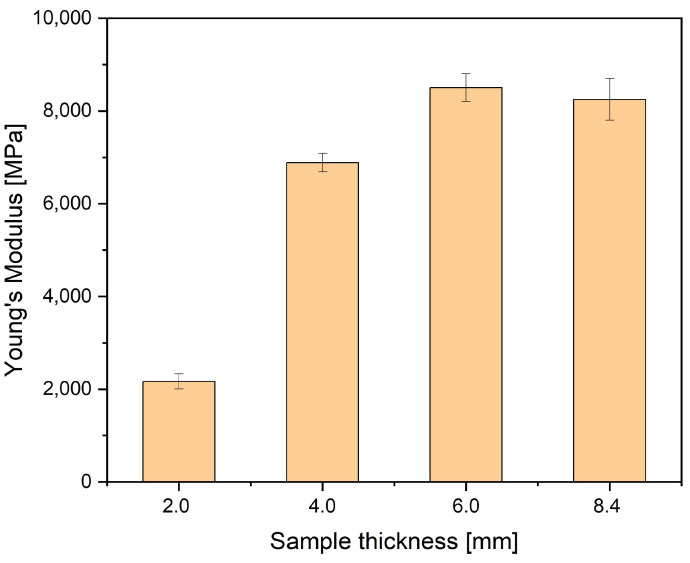
Young’s modulus for four samples thicknesses. Values are 2167 MPa, 6888 MPa, 8500 MPa, and 8250 MPa for 2 mm, 4 mm, 6 mm, and 8.4 mm thick samples, respectively.

Strength properties complement extensive structural studies of porous polyamide composites reinforced with glass fiber. Thick-walled 6- and 8.4 mm samples are characterized by a very high value of tensile strength (R_m_) and Young’s modulus (E) determined in the tensile test (Figure 17 and Figure 18). 

The highest strength and Young’s modulus for 6 mm thick moldings are caused by the smallest gas pores in the structure of these moldings. The high flow rate of the polymer melt caused the small pore size for these samples. Wang [30] and his team showed that changing the injection speed can cause significant changes in the mechanical properties of molded parts produced using microcellular injection molding technology. Błędzki et al. conducted similar tests [46].

Likewise, in the case of samples with a thickness of 4 mm, Young’s modulus is higher than stated in the datasheet of the material supplier. Also, nitrogen influenced the results, thanks to which thick-walled moldings without sink marks were obtained. Moreover, the injection point location, the cavities’ shape, and the gate type resulted in a laminar flow and the proper filling of the cavities, which caused an advantageous arrangement of the short fibers in the melt flow direction in a significant sample volume. It was previously found that the skin’s thickness increased with the moldings’ increasing thickness. The above-described effects resulted in producing a multilayer composite in which the unfoamed skin layer determined the value of Young’s modulus and tensile strength.

**Figure 19:**
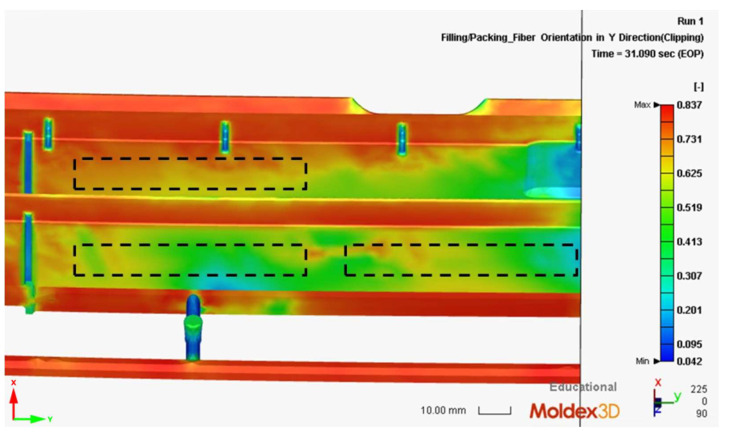
Glass fibers orientation simulated in Moldex3D^®^ software, along with cutting the samples for mechanical testing, marked by dashed lines. The color scale shows fibers orientation, where 1 and 0 values mean parallel and perpendicular orientation to the y-axis (testing tensile force).

Another factor contributing to the strength properties of foamed samples is that the size of the gas pores in the core and transition zone is small compared to other papers. The results for a sample with a thickness of 2 mm significantly differ from the others; however, producing them from a different type of polyamide is not the reason. For PA6 GF30, the supplier’s data sheet is very similar to PA66 GF30. Young’s modulus is the same, while stress at break is 110 MPa and 100 MPa, respectively. The different locations of injection points in relation to the places of sampling from a large-size molded part cause a significant decrease in E and R_m_. The simulation analysis showed that, in these places, the fibers are mostly arranged perpendicular to the stretching direction of the samples (Figure 19). It was the reason for such a drastic drop in the value of both parameters. Because of this part’s geometry, it was impossible to take these samples from other places of the large-sized moldings, primarily because of the ribs and thickness differences.

**Figure 20:**
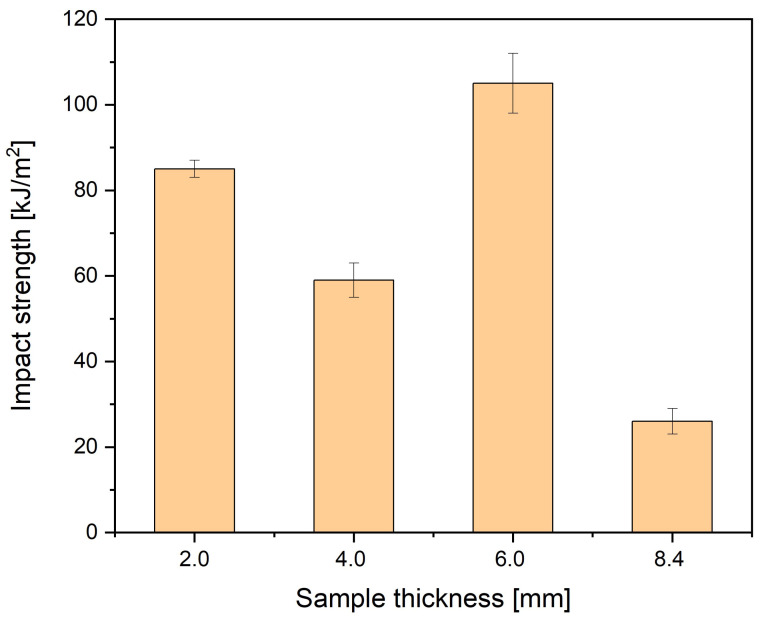
Impact strength for four samples thicknesses. Values are 85, 59, 105, and 26 kJ/m^2^ for 2 mm, 4 mm, 6 mm, and 8.4 mm thick samples.

The increasing thickness of foamed thick-walled moldings of PA66 GF30 improved its impact strength. The 8.4 mm thick samples needed to be notched since they did not break, even when using the 50 J pendulum. Therefore, it is not possible to compare these results to the others (Figure 20). 

For a sample with a thickness of 6 mm, a much better impact strength was obtained compared to the producer’s material data sheet (65 kJ/m^2^). Also, for 8.4 mm thick, notched samples, the impact strength is about four times higher than in the producer’s characteristic (6 kJ/m^2^). These beneficial results for thick-walled samples are due to the synergistic interaction of two effects: good nitrogen pore distribution and their small dimensions and a much larger cross-section of samples with a significant solid skin thickness. With the 2 mm thick sample, the impact strength was 25% lower than that of the solid PA6 GF30 (110 MPa). The core part had non-uniform pores with significantly larger dimensions, resulting in the decrease in impact strength. They behaved as structural notches during impact loading, causing the brittle fracture. This effect was limited by the fibers’ heterogeneous arrangement in the volume of samples cut from the large-size molding.

## 4. Conclusions

The research showed a strong, yet non-linear correlation between molding thickness and geometry and pore size and distribution. The most advantageous pore size and distribution from the mechanical properties point of view was observed for 6 mm thick PA glass-fiber-reinforced moldings (Table 4). The pore size of thick PA66 GF30 molded parts is smaller than that reported by other authors for thinner samples. This favorable pore size distribution in 6 and 8.4 mm thick-walled moldings had a significant impact on their significant mechanical properties. 

Despite differences in the values of the pore size and distribution results obtained using different methods, the commercial X-ray computed tomography method used in the presented article was positively verified. The largest deviations were observed in the simulation results, which may help adapt the FEM model for thick-walled moldings (above 4 mm thickness) obtained using the MIM method.

Such complex (three-phase) structures with pore size diameters even below glass fiber diameter are challenging to investigate with commercial X-ray computed tomography and demand properly performed scanning, selecting parameters, resolution, and skillful data processing. The 3D data analysis results are very useful; however, it is still worth comparing them with SEM images for the same samples.

The results of the conducted research may be the basis for encouraging the greater use of porous structures reinforced with short fibers in thick-walled elements (typical mechanical increase in strength by increasing the cross-section in real products). It is especially possible since obtained thick wall moldings do not have deformations and sink marks on the surface. Therefore, industries can apply microcellular thick-walled pieces as an alternative to aluminum alloys, enabling them to bear high loads.

Increasing the use of lightweight polymeric moldings reduces the carbon footprint and provides the possibility of secondary processing, closing the product life cycle, which fulfills the demand of a circular economy. 

## Figures and Tables

**Table 1:** Samples analyzed in the presented article.

Sample Thickness, mm	Material	Density Reduction, %
2	PA6 30GF (Akulon K224-HG6) (DSM, Heerlen, The Netherlands)	5.1
4	PA66 30GF (Technyl AR 130GF) (Solvay, Brussels, Belgia)	5.5
6	PA66 30GF (Technyl AR 130-1) (Rhodia, La Defence, France)	5.3
8.4	PA66 30GF (Technyl AR 130-1) (Rhodia, La Defence, France)	5.3

**Table 2:** General information about the materials used in the research.

Sample Thickness, mm	Density, kg/m³	MFI, g/10 min	Tensile Modulus, MPa	Stress at Break, MPa	Charpy Impact Strength, kJ/m^2^
2	1350	30	6000	110	110
4	1370	34	6000	95	65
6	1370	34	6300	95	35
8.4	1370	34	6300	95	35

**Table 3:** Injection molding processing parameters applied to produce samples of different thicknesses in MuCell^®^ technology.

IM Parameters	2 mm	4 mm	6 mm	8.4 mm
filling pressure, MPa	112.9	70	70	70
melt temperature, °C	285	285	285	285
holding pressure, MPa	20	16	14	14
holding time, s	0.3	0.3	0.3	0.3
mold temperature, °C	90	90	90	90
cooling time, s	28	30	50	50

**Table 4:** Mesh parameters used for simulations conduction with the use of the Moldex3D^®^ software.

Parameter	2 mm	4.2 mm	6 mm + 8.4 mm
Solid mesh elements	1,038,216	78,387	141,605
Surface mesh elements	196,920	14,162	26,806
Mesh size (mm)	2.72	2.475	2.475
Mesh boundary layers	3	3	3

**Table 5:** Thickness of a skin layer and average pore size measured by SEM, by X-ray microtomography, and simulated by Moldex3D^®^ 2022 software for four different samples thicknesses.

Sample Thickness, mm	Skin Layer Thickness (by SEM), mm	Avr. Pore Size (by SEM), µm	Avr. Pore Size (by µCT), µm	Pore Size in the Core Part (by Moldex3D^®^), µm
2	~0.5	56.21 ± 10.61	32.59	74.21
4	~0.7	18.41 ± 9.06	28.37	71.44
6	~0.8	15.73 ± 4.13	16.87	44.59
8.4	~1.2	17.98 ± 5.08	23.95	60.28

## Data Availability

The data supporting this study’s findings are available from the corresponding author (Piotr Szewczykowski) on request.
